# Protocol for isolation of interstitial fluid from tumor-draining lymph nodes for proteomic profiling in murine breast cancer models

**DOI:** 10.1016/j.xpro.2026.104816

**Published:** 2026-09-01

**Authors:** Greta Mattavelli, Jonathan J. Swietlik, Moutaz Helal, Arpa Aintablian, Felix Meissner, Angela Riedel

**Affiliations:** 1 Mildred Scheel Early Career Centre (MSNZ) for Cancer Research Würzburg, University Hospital Würzburg and University of Würzburg, Würzburg, Germany; 2 Experimental Systems Immunology, Max Planck Institute of Biochemistry, Martinsried, Germany; 3 Institute of Innate Immunity, Department of Systems Immunology and Proteomics, Medical Faculty, University of Bonn, Bonn, Germany; 4 Theodor Boveri Institute, Department of Biochemistry and Molecular Biology, Biocenter, University of Würzburg, Würzburg, Germany

**Keywords:** Cancer, Cell Biology, Immunology, Proteomics

## Abstract

Tumors release soluble factors that reach tumor-draining lymph nodes (TDLNs), where they induce remodeling already at the pre-metastatic stage and alter the local proteomic landscape. Here, we describe a protocol for isolating interstitial fluid from primary tumors and TDLNs in murine breast cancer models to study extracellular protein composition while preserving tissue viability and integrity. We outline key steps for tissue preparation, fluid extraction, and downstream bioinformatic analysis to support the characterization of tumor-associated changes in TDLNs.

For complete details on the use and execution of this protocol, please refer to Mattavelli et al.^1^

## Highlights

• Steps for orthotopic inoculation and tissue collection in murine breast cancer models
• Protocol for interstitial fluid isolation from lymph nodes and primary tumors
• Steps for preparing interstitial fluid samples for mass spectrometry-based proteomics
• Guidance on downstream proteomic data analysis and visualization

## Abstract (editor highlights)

Publisher’s note: Undertaking any experimental protocol requires adherence to local institutional guidelines for laboratory safety and ethics.

## Graphical abstract

**Figure:**
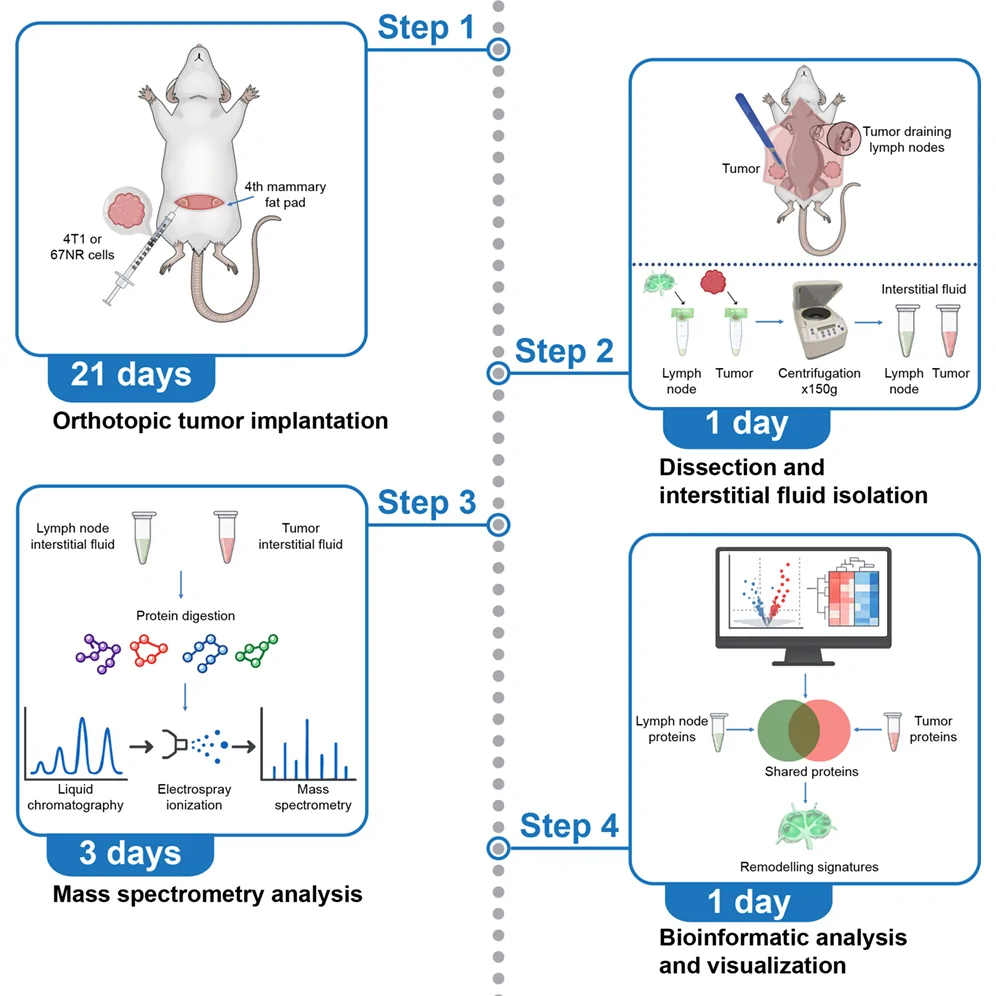

## Before you begin

Tumor-draining lymph nodes (TDLNs), localized downstream of the tumor, undergo remodeling during breast cancer progression. Many factors, including metabolites, proteins, and extracellular vesicles, are either drained to or induced within TDLNs by the primary tumor.

This protocol provides a standardized protocol to analyze and quantify the extracellular proteins that are secreted or induced by the primary tumor. It involves isolating the interstitial fluid (ISF) from primary tumors and TDLNs and performing downstream proteomic analysis.

It is important to investigate the primary draining lymph node (LN) of the tumor model prior to any analysis. This protocol was optimized for bilateral breast tumor cell injections into the 4^th^ mammary fat pad. However, unilateral injections can also be chosen as a model to study, pending the overall aim. These tumors classically first drain to the inguinal LN but later to the axillary LN, which is analyzed herein.^1^ Thus, time points after tumor cell inoculation need to be carefully considered. The protocol has been established in 4T1 and 67NR breast tumor models in BALB/c mice but can be extended to additional models, such as E0771 or AT3 in C57BL/6 mice. Although certain proteomic features may be conserved across TDLNs from different models, others are likely to be model-specific. This protocol uses female mice because breast cancer mostly affects women. As sex is a known biological variable, users adapting this protocol to models relevant to both sexes should include sex as a variable in design and interpretation.

The ISF isolation procedure for LNs can readily be applied to other disease contexts, including infectious conditions. Moreover, it can be adapted for the analysis of proteins in the ISF of patient-derived LN samples (data not shown). In addition, this protocol can be used for metabolomic analysis, either in combination with or instead of proteomic analysis.

### Innovation

Investigating the extracellular compartment is performed via several methods: whole-tissue lysates, in vivo microdialysis, and plasma profiling. Each of these methods had inherent limitations, such as the inability to separate secreted from intracellular proteins, being technically demanding and yielding low protein amounts, or only reflecting systemic rather than tissue composition, respectively. Centrifugation-based ISF isolation has been described for bulk solid tumors, largely in the context of metabolomics.^2^^,^^3^

This protocol’s innovative potential lies in adapting low-speed centrifugation to LN and specifically to TDLN at a pre-metastatic stage. It presents a unified workflow, which enables comprehensive protein characterization in ISF, while maintaining tissue viability, integrity, and compatibility with downstream analyses. Because centrifugation is performed under conditions where cell viability remains unaltered and tissue architecture is preserved, the same tissue remains available for downstream analyses like flow cytometry, single-cell RNA-sequencing, and immunofluorescence, enabling paired extracellular-proteome and cellular readouts from a single sample.

Furthermore, given its high translational potential, after optimization and careful consideration, this protocol can serve as a template for ISF isolation from other cancer types, tissues, and patient-derived material, as well as be combined with other downstream analyses, such as metabolomics.

### Institutional permissions

All experiments were designed to minimize the number of euthanized animals, in compliance with the 3Rs (replacement, reduction, and refinement) principle. Female immunocompetent BALB/c experimental mice (aged 6–8 weeks) were purchased from Envigo laboratory and acclimated to the animal facility for one week before the start of experiments. Mice were housed in conventional facilities in individually ventilated cages, under a 12-hour light-dark schedule at 22°C and in the presence of 2–4 cage mates. Standard autoclaved laboratory diet and water were provided ad libitum.

All animal studies were approved by the governmental review board of the state of Bayern, Regierung von Unterfranken, and according to the German legal regulations (License number: 55.2 2532-2-1242). Humane endpoints were defined according to institutional guidelines, and animals were euthanized if tumor burden (>0.52 cm^3^) exceeded approved limits or if signs of distress were observed. Animals were monitored daily post-surgery for changes in posture, wound appearance, body weight, fur condition, body condition score, signs of pain (grimace scale), and mobility until the end of the experiment.

Acquire all necessary permissions from the relevant institutions before starting the experiments.

### Injection into the 4^th^ mammary fat pad

**Timing: Variable**

The first preparation is to become proficient in the orthotopic injection of murine breast cancer cells into the 4^th^ mammary fat pad. Although the surgical procedure is described below, adequate training and technical competence are essential before proceeding with ISF isolation and subsequent downstream experiments to ensure reliable and reproducible results. Please note that training and performance of this technique may be subject to institutional and governmental regulations. All necessary ethical approvals and permissions must be obtained prior to conducting the procedure.

### Isolation of the axillary lymph node

**Timing: Variable**

The second preparation is to become comfortable with isolating the axillary LN. Draining the 4^th^ mammary fat pad, the inguinal LN will be the primary TDLN. However, as the tumor grows to about 500 mm^3^ size, it will engulf this area. At this stage, the axillary LN becomes the main TDLN, which can be confirmed by increased cellularity and remodeling of immune and stromal cell populations.^1^^,^^4^ At the endpoint used in this protocol, the axillary LN is collected as a pre-metastatic TDLN and should be isolated consistently across experimental groups. Axillary LNs can be difficult to identify. Therefore, it is advisable to practice isolating axillary LNs from non-tumor-bearing mice postmortem before proceeding with this protocol.

1. Secure the mouse’s paws to the dissection table using needle pins.
2. Make a vertical incision through the skin from the lower abdomen to the neck. Then, make two straight cuts pointing toward the forelimbs.
3. The axillary LNs are located in the axillary region of the mouse and are not immediately visible once the mouse is opened.
4. Use fine-tip forceps to precisely remove the LN.
5. Carefully remove the connective tissue and fat until the axillary LNs become visible. The LNs are small, pearl-colored, oval-shaped structures.
6. Gently remove the LN in one piece, as the area is highly vascularized. Rupturing blood vessels may cause bleeding that obscures the tissue and complicates identification.

### Optimization of experimental design

The experimental groups consisted of at least three mice per condition, with each mouse serving as one biological replicate. Researchers adapting this protocol should determine the appropriate number of mice per group by performing a sample-size calculation tailored to their specific comparison. For the proteomic analysis, left and right axillary LNs from the same mouse were pooled and treated as one biological replicate.

When planning experiments, include additional animals beyond the minimum required to account for potential sample loss, insufficient ISF yield, or exclusion due to biological variability. Always include naïve axillary LNs from non-tumor-bearing mice and process them in parallel as controls. Non-metastatic LNs may also serve as controls (e.g., those collected following unilateral tumor cell injection); however, this approach is not employed in the presented protocol. Furthermore, within this protocol, pre-metastatic TDLNs are studied. In tumor-bearing mice (4^th^ mammary fat pad injection), axillary TDLNs are thus collected at a pre-metastatic stage. LNs are classified as pre-metastatic based on the absence of detectable metastatic cells at the time of collection, which is confirmed by us using GFP-labeled tumor cells in combination with flow cytometry. Alternative methods, such as immunofluorescence or quantitative PCR for tumor-specific markers, are equally suitable for this purpose.^4^ Importantly, LN metastatic status should be determined prior to ISF analysis, as this classification has significant implications for downstream data interpretation. At the experimental endpoint used here, axillary TDLNs showed tumor-induced remodeling but did not contain detectable tumor cells, consistent with the pre-metastatic status described in Mattavelli et al.^1^

Samples from different experimental groups were processed in parallel whenever possible

Samples were excluded only in cases of failed orthotopic injection (including peritoneal tumor growth) or absence of tumor formation or insufficient ISF yield for downstream proteomics. To ensure comparability across experimental groups, tumor size at endpoint was required to be within a predefined range (±20% of the target volume, around 500 mm^3^). This minimized variability associated with tumor burden and enabled consistent proteomic profiling across conditions.

## Key resources table

**Table:** 

REAGENT or RESOURCE	SOURCE	IDENTIFIER
**Chemicals, peptides, and recombinant proteins**		

Fetal Bovine Serum (FBS)	Sigma Aldrich	Cat# F7524
Penicillin-Streptomycin (10,000 U/mL)	Thermo Fisher Scientific	Cat# 15140122
DMEM, high glucose, pyruvate	Thermo Fisher Scientific	Cat# 41966052
Trypsin-EDTA solution	Sigma Aldrich	Cat# T4049
Trypan blue	Thermo Fisher Scientific	Cat# T10282
Corning® Matrigel® Growth Factor Reduced (GFR)	Corning	Cat# 354230
Phosphate buffered saline (PBS)	Sigma Aldrich	Cat# P2272
Buffer A: Water with 0.1% Formic Acid (v/v), Optima™ LC/MS Grade	Fisher Scientific	Cat# LS118-500
Buffer B: 80% Acetonitrile, 20% Water with 0.1% Formic Acid, Optima LC/MS	Fisher Scientific	Cat# LS122500
Pierce™ Premium Grade TCEP-HCl	Thermo Fisher Scientific	Cat# PG82089
2-Chloroacetamide	Sigma Aldrich	Cat# 22790-250G-F
Water, Optima™ LC/MS Grade	Fisher Scientific	Cat# W6500
Empore™ SPE Disks (SDB-RPS)	Sigma Aldrich	Cat# 66886-U
Trypsin/Lys-C Mix, Mass Spec Grade	Promega	Cat# V5071
Isoflurane - Iso-Vet 1000 mg/g	Dechra	N/A
Metamizole HEXAL® 500 mg/ml	Hexal	N/A
Bepanthen® Eye and Nose Ointment	Bayer	N/A
Octenisept ® Aqueous wound and mucous membrane antiseptic	Schülke	N/A
Zombie Violet Fixable Viability Dye	BioLegend	Cat# 423106

**Deposited data**		

Mass spectrometry proteomics data	Mattavelli et al.^1^	PRIDE: PXD066773
R script template for downstream analysis	This paper	Zenodo: https://doi.org/10.5281/zenodo.21293916.

**Experimental models: Cell lines**		

Mouse: 67NR cell line (*Mus musculus*, female, BALB/c mammary tumor–derived)	Fred Miller, Karmanos Cancer Institute/Wayne State University, Detroit, MI, USA^5^	RRID:CVCL_9723; no commercial alternative identified
Mouse: 4T1 cell line (*Mus musculus*, female, BALB/c mammary tumor–derived)	Fred Miller, Karmanos Cancer Institute/Wayne State University, Detroit, MI, USA^5^	RRID:CVCL_0125; also available from ATCC (Cat# CRL-2539)

**Experimental models: Organisms/strains**		

Mouse: BALB/c OlaHsd, female, wild-type, 6–8 weeks old, (*Mus musculus*)	Envigo Laboratory	Cat# 16205F

**Recombinant DNA**		

Episomal GFP	Matthias Bozza, DNA Vector Laboratory, DKFZ Heidelberg, Germany^6^	N/A

**Software and algorithms**		

GraphPad Prism (version 8.1.0)	GraphPad software	https://www.graphpad.com/features
R (4.3.2)	The R Foundation	https://www.r-project.org/
RStudio (2023.12.1+402)	RStudio	https://posit.co/
ggplot2 R package (3.3.6)	Wickham et al.	https://ggplot2.tidyverse.org
EnhancedVolcano R package (1.22.0)	Blighe K et al.	https://bioconductor.org/packages/release/bioc/html/EnhancedVolcano.html
Umap R package (0.2.10)	McInnes and Healy	https://github.com/tkonopka/umap
RColorBrewer (1.1–3)	Cran.r-project	https://cran.r-project.org/web/packages/RColorBrewer/index.html
DIA-NN (1.8)	Demichev V et al.	https://github.com/vdemichev/diann
Xcalibur (4.4.16.14)	Thermo Fisher Scientific	N/A

**Other**		

Invitrogen Countess automatic cell counter	Thermo Fisher Scientific	Cat# A49862
BD Medical™ BD Micro-Fine™ Insulin Syringe, 29G	BD	Cat# 324892
7 mm Reflex Wound Closure System	Reflex	Cat# RF7 CS
7 mm Reflex Applier	Reflex	Cat# RF7 APL
Iris Scissors, 9 cm, curved	Fine Science Tools	Cat# 14061–09
Dumont #55 Forceps	Fine Science Tools	Cat# 11255–20
Dumont #7 Forceps	Fine Science Tools	Cat# 11271–30
Whatman® quantitative filter paper, ashless, Grade 40	Merk	Cat# WHA1440042
KERN analytical balance ABS-N/ABJ-NM	KERN	N/A
Sealing film PARAFILM	PARAFILM	N/A
ThermoMixer ® C	Eppendorf	Cat# 5382000015
Isoflurane anesthesia system	Groppler	N/A
Protein LoBind® Tubes, 1.5 mL	Eppendorf	Cat# 0030108116
NanoDrop One spectrophotometer	Thermo Fisher Scientific	Cat# ND-ONE-W
Centrifuge 5424 R	Eppendorf	Cat# 5404000010
Centrifuge 5810/5810 R	Eppendorf	Cat# 022627082
pluriStrainer Mini cell strainer, mesh size 20 μm	pluriSelect	Cat# 43–10020–50
Concentrator plus	Eppendorf	Cat# 5305000509
EASYnLC 1200 System	Thermo Fisher Scientific	N/A
Orbitrap Exploris™ 480 Mass Spectrometer	Thermo Fisher Scientific	N/A

## Materials and equipment

**Table:** Supplemented DMEM

Reagent	Final concentration
DMEM	N/A
FBS	10%
Penicillin-streptomycin	1%

***Note:*** Prepare supplemented DMEM in a sterile environment and use the medium within four weeks. Store it at 4°C.

**Table:** 5× reduction/alkylation master mix

Reagent	Final concentration
Pierce™ Premium Grade TCEP-HCl	10 mM
2-Chloroacetamide	30 mM
Tris-HCl pH 8.5	100 mM

***Note:*** Prepare master mix fresh immediately before use and keep it protected from light at 21°C. Do not store or reuse excess master mix.

## Step-by-step method details

### Preparation of breast cancer cells

**Timing: 1 h**

Here, we describe how to prepare, count, and resuspend murine 4T1 and 67NR breast cancer cells for orthotopic injection into the 4^th^ mammary fat pad.

1. Maintain murine 4T1 or 67NR breast cancer cells in supplemented DMEM at 37°C with 5% CO_2_ in a humidified incubator, under sterile conditions.

***Note:*** Avoid reaching full confluence; passage cells at 80–90% confluence and keep the sub-cultivation ratio at 1:10. Passage the cells twice before injecting them into mice and avoid using cells at high passages.

2. Remove the cell culture flasks from the incubator and rinse with pre-warmed PBS under a sterile hood.
3. Detach the cells with pre-warmed (to 37°C) 0.25% Trypsin-EDTA solution in a 37°C incubator.
4. Stop the trypsin digestion by adding an excess of fresh culture medium and transfer the cells into a 15 mL conical tube.
5. Count cells to determine the concentration and assess viability using trypan blue (method variable). Cells should be at least 80% viable to ensure optimal engraftment.

***Note:*** Use an automated cell counter to reduce variability among experiments and operators, and to improve reproducibility in tumor growth.

6. Pellet the appropriate number of cells for injections by centrifugation for 5 min at 300 × *g* at 4°C.
7. Resuspend the pellet in PBS to wash residual medium off and pellet again by centrifugation (5 min, 300 × *g*, at 4°C).
8. Resuspend the cell pellets:
  a. For 4T1: at a final concentration of 1 × 10^5^ cells/mL (5,000 cells in 50 μL per mammary fat pad injection), in cold PBS.
  b. For 67NR: at a final concentration of 2 × 10^6^ cells/mL (100,000 cells in 50 μL per mammary fat pad injection), in a 1:1 mixture of cold PBS and Matrigel.

***Note:*** When calculating the number of cells needed, account for the fact that injections are performed bilaterally. Also, to allow for loss during syringe preparation (dead volume) and pipetting, prepare double the volume of cell suspension required.

9. Store the cell suspensions on ice until injection into mice.

***Note:*** These cell numbers have been optimized to match tumor volume (500 mm^3^) at the endpoint between different mouse models around day 21 after injection.

**CRITICAL:** Inject the tumor cells within 1 h of resuspension to avoid a decrease in viability. If injecting more than 8 mice, prepare the cell suspensions in consecutive batches to ensure optimal viability.

### Orthotopic injection into the 4^th^ mammary fat pad and tumor growth

**Timing: 2 h for breast cancer cell injection and 21 days after injection for end-stage tumors**

This section describes the process of orthotopically injecting breast cancer cells into the 4^th^ mammary fat pad of mice, followed by monitoring tumor growth until the experimental endpoint.

10. Mouse preparation:
  a. Use healthy female BALB/c mice that are 6–8 weeks old.
  b. Shave the area of the lower abdomen at the height of the 4^th^ mammary glands to expose the abdomen and injection sites.

**CRITICAL:** Anesthesia and analgesia must only be administered by trained personnel according to the approved animal license. Monitor anesthesia depth and respiration throughout the procedure and ensure that appropriate ventilation is in place to minimize exposure of personnel.

**Figure 1:**
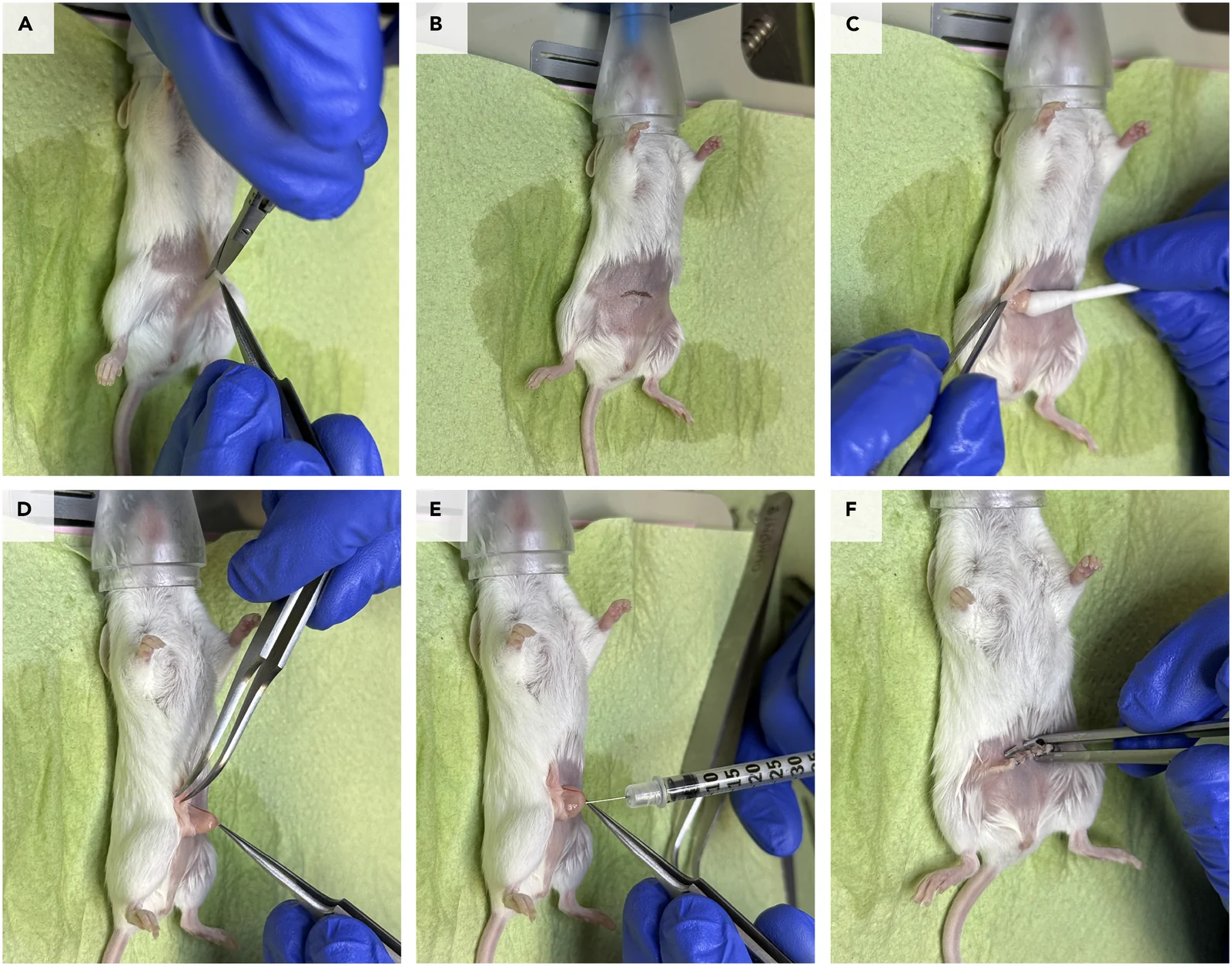
Workflow for orthotopic injection in the 4^th^ mammary fat pad (A) Skin incision of the belly at the level of the 4^th^ mammary fat pad. (B) Appearance of the incision before separation of the mammary fat pad from the skin. (C) Separation of the skin from the mammary fat pad using a sterile cotton swab. (D) Exposure and positioning of the mammary fat pad for injection. (E) Injection of the tumor cell suspension into the mammary fat pad using a 29G insulin syringe. (F) Closure of the skin incision using surgical clips.
  b. Use a sterile cotton swab wetted with alcohol-free disinfectant to dissociate the skin from the right mammary fat pad (Figure 1C).
  c. Gently resuspend the prepared cell suspension and load a 29G insulin syringe with 100 μL.
  d. Use forceps to hold the mammary fat pad.
    i. Insert the syringe and slowly inject the cells to form a visible bubble within the mammary fat pad (Figures 1D and 1E).
    ii. After the injection, carefully pull out the needle while still pressing the plunger.
  e. Repeat the same procedure on the left side.
    ***Note:*** If the cell suspension leaks, use a sterile cotton swab to absorb the spilled liquid to reduce the risk of non-orthotopic tumor formation.
  f. After injecting both mammary fat pads, close the skin incision using surgical clips (Figure 1F).
  g. Remove the animal from the anesthesia station.
  h. Apply alcohol-free disinfectant and monitor the animal until fully awake.
  i. After surgery, monitor animals daily, or as often as indicated in the animal license.
    ***Note:*** Monitoring should include: posture, wound appearance, body weight, fur condition, body condition score, signs of pain (grimace scale), and mobility, and be continued until the end of the experiment.
13. Tumor growth:
  a. Use a caliper to measure tumor growth 3 times per week from day 5 until the endpoint is reached. Measure the width and the length of each tumor.
  b. To calculate tumor size, apply the ellipsoid formula π/6 × length × width.^2^

***Note:*** Ensure compliance with the regulations of your animal license for all steps in this section.

11. Mouse analgesia and anesthesia:
  a. Administer subcutaneous analgesia, metamizole, 1 h prior to the start of surgery (as indicated in the animal license).
  b. Place the mouse in an isoflurane chamber (2 L/min of oxygen, 3.5% of isoflurane).
  c. Confirm anesthesia by the absence of paw withdrawal reflex and move the mouse to a nose mask anesthesia system (2 L/min of oxygen, 2.5% of isoflurane).
  d. Apply ophthalmic ointment to prevent eye dryness.
12. Cancer cell inoculation:
  a. Use fine-tip scissors to create a small incision in the abdominal skin at the level of the 4^th^ mammary fat pad (Figures 1A and 1B).
    ***Note:*** Always use sterile instruments to lower the risk of infections.

### Isolating and processing of mouse tissues

**Timing: 2 h**

The postmortem processing of mouse TDLNs and primary tumors is described in this section. It outlines the dissection procedures and handling necessary to preserve tissue integrity for subsequent ISF extraction. Continue these steps once the tumors have grown to the experimental endpoint or targeted size.

***Note:*** Use tumors of comparable size across experimental groups to improve consistency and comparability of downstream analyses.

14. Euthanize the mice using an approved method of your animal license, such as cervical dislocation or CO_2_ inhalation.
15. Pin the mouse down with needle pins at all four paws.
16. Sterilize the surface of the mouse by applying 70% alcohol to reduce potential contamination during tissue isolation.

***Note:*** Clean and sterilize instruments after each sample, or replace them with sterile instruments, to prevent sample carryover.

**Figure 2:**
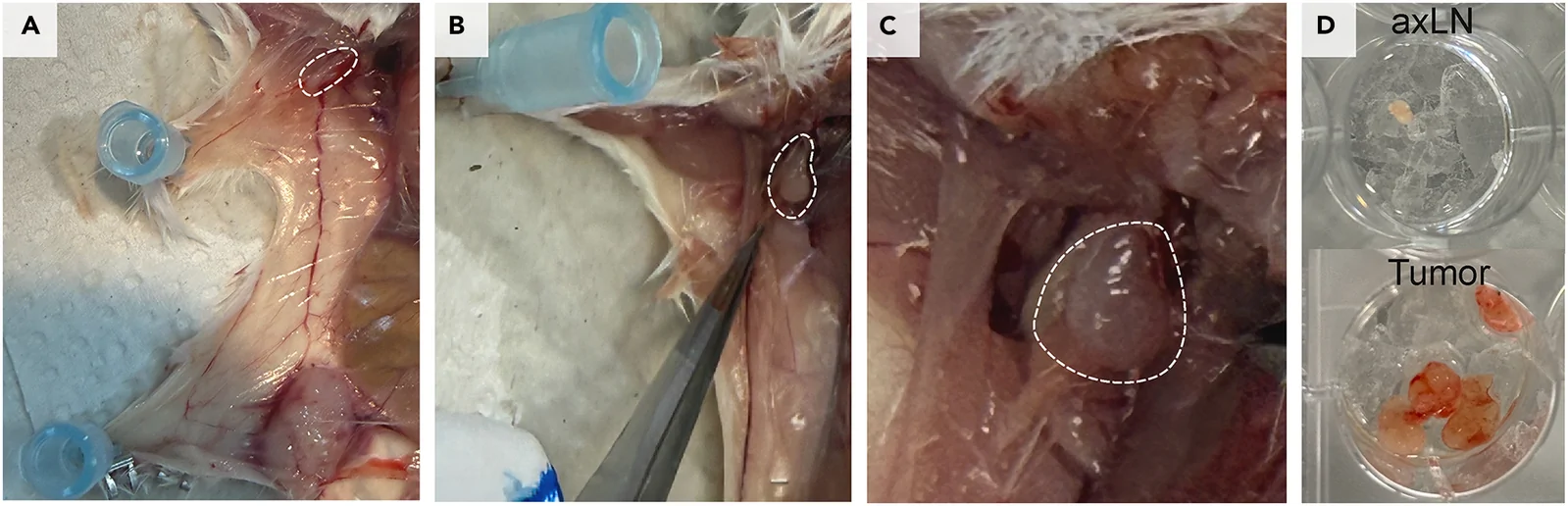
Overview of axillary lymph node and tumor collection (A) Mouse positioning and exposure of the primary tumor and the axillary region for lymph node collection. The dashed outline indicates the anatomical location of the axillary lymph node (axLN). (B) Identification and dissection of the axLN using fine-tip forceps. The dashed outline indicates the axLN. (C) Magnified view of the axLN, with the dashed outline indicating its position. (D) Collected axLN and tumor stored in a 12-well plate on ice before ISF isolation.
18. Dissect the primary tumors and the axillary LNs (TDLNs in this model) using fine-tip forceps, as these facilitate precise LN dissection (Figures 2B and 2C).

***Note:*** Handle the tissues carefully to minimize damage. As LNs are embedded within fat tissue, carefully dissect and remove surrounding fat to avoid contamination of the sample. Variability in LN dissection and processing time between operators may affect ISF yield and composition, consistent training and standardized workflow timing are essential.

17. Create a midline skin incision extending from the lower abdomen to the neck. Then, make two perpendicular incisions extending toward the forelimbs (Figure 2A).

**CRITICAL:** Axillary LNs can be difficult to identify as their anatomical location limits their visibility. Opening the mouse correctly, stretching the tissue, and using good light aids in this process. Use a stereomicroscope for dissection if needed.

19. Add ice-cold, sterile PBS to a 12-well plate and use it to wash the dissected organs, removing any blood or debris. Always keep the samples on ice (Figure 2D).
20. Dry the tissues gently on Whatman paper.
21. Use a precision scale to weigh each tissue and record the weight for subsequent yield assessment and data interpretation.

***Note:*** LNs have very low weight, therefore, accurate weighing is essential, as it is critical for downstream processing and contextual interpretation of ISF yield.

22. After weighing, return the samples to a 12-well plate, and keep on ice until the next step.
23. For each mouse, record the following data:
  a. Tumor volume and weight at harvest.
  b. LN identity (TDLN or naïve LN) and weight.
  c. ISF volume yield.

**CRITICAL:** To ensure the maximum yield of ISF while preserving high tissue viability, harvest and process one mouse at a time. The time between dissection and centrifugation should not exceed 15 min.

### ISF isolation of TDLNs and primary tumor by centrifugation

**Timing: 30 min**

This section outlines the isolation of ISF from collected tissues (TDLNs and primary tumor) using low-speed centrifugation.

**Figure 3:**
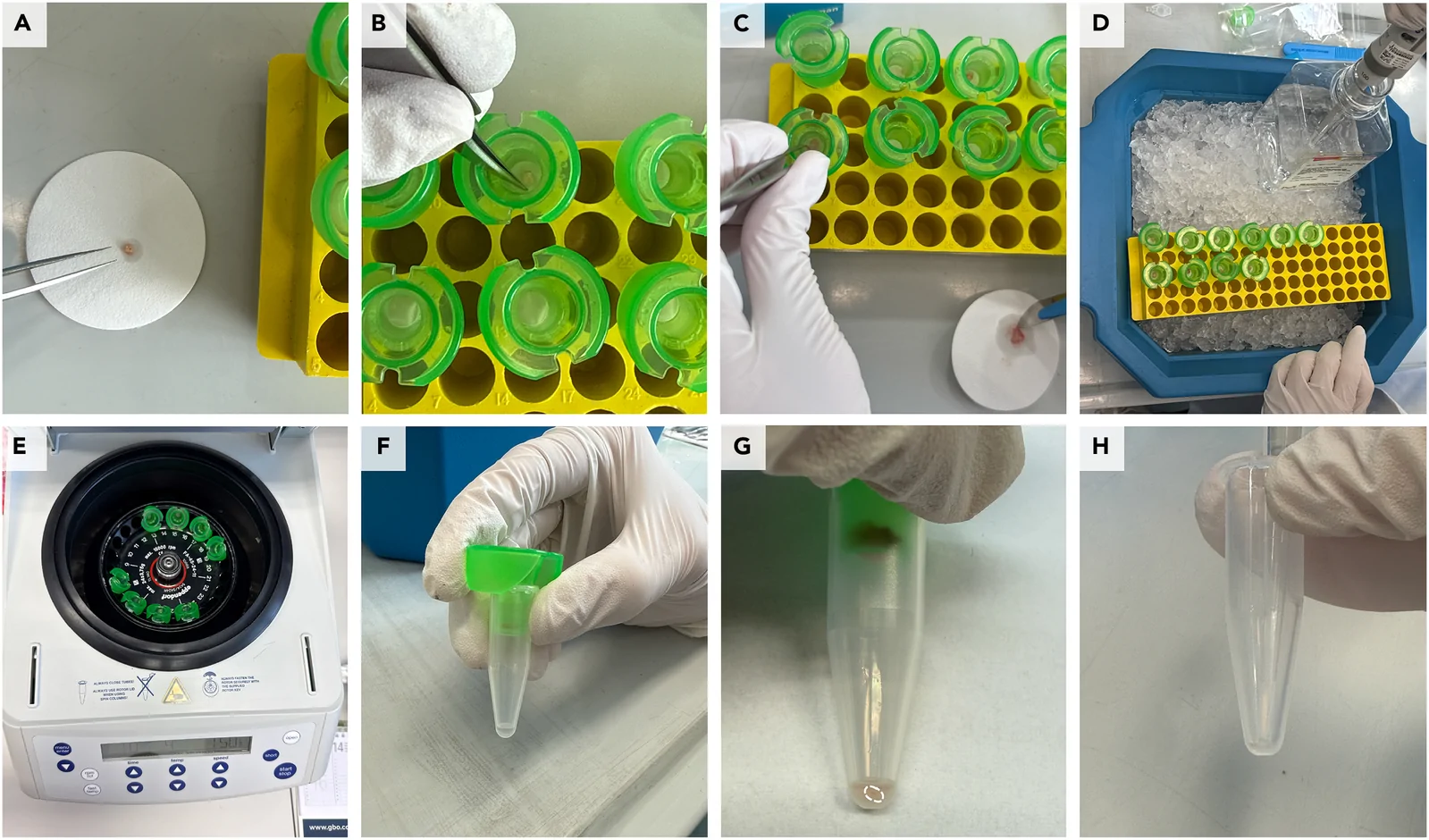
Step-by-step ISF extraction (A) Drying of lymph node tissue on Whatman paper. (B) Placement of lymph node tissue on a 20 μm strainer fitted onto a 1.5 mL tube. (C) Cutting and drying of tumor tissue on Whatman paper, followed by placement of tumor pieces on a 20 μm strainer. (D) Addition of 10 μL ice-cold PBS to the tissue and maintenance of samples on ice before centrifugation. (E) Centrifugation of the samples at 150 × g for 10 min at 4°C. (F) Appearance of the sample after centrifugation, with tissue retained on the strainer and liquid collected at the bottom of the tube. (G) Magnified view showing separation of the ISF fraction from the sedimented material. (H) Recovery of the ISF fraction without disturbing the sedimented material.
26. Position the tissue on the cell strainer and add 10 μL of cold sterile PBS on top (Figures 3B–3D).
  a. For LNs: keep the tissue intact as it has a small size (Figures 3A and 3B).
  b. For tumors: cut them into 50 mg pieces, as they are larger in size, to ensure better extraction of ISF (Figure 3C). E.g., for a standard tumor of around 500 mg, cut it into 10 pieces.

***Note:*** Naïve LN ISF volume is insufficient for recovery (1–2 μL for each LN), so a fixed volume of 10 μL PBS is added uniformly to all LN samples regardless of LN size. Therefore, samples should be processed quickly to minimize evaporation-related loss. Recovered ISF represents diluted extracellular fluid, so only compare samples that have been processed identically.

24. Prepare 1.5 mL tubes by cutting the lid and placing a 20 μm strainer on top of each tube.
25. Remove the tissue from the plate and gently dry it on Whatman paper (Figure 3A).

27. Centrifuge at 150 × *g* for 10 min at 4°C (Figure 3E).

**CRITICAL:** The centrifugation speed was optimized to maximize ISF recovery while minimizing tissue disruption and release of intracellular proteins. This step was adapted from previously published protocols.^2^^,^^3^

***Note:*** All centrifugation steps should be performed at 4°C to minimize protein degradation and maintain sample integrity.

28. Gently aspirate the ISF (supernatant) without disturbing the sedimented material at the bottom of the tube, which may contain cellular debris and small cells (Figures 3F and 3G).

***Note:*** As a quality control step, ISF purity was assessed by flow cytometry. ISF was stained with a viability dye to identify cellular contamination. High-quality ISF preparations contained <1% of cells of total events and minimal cellular debris.

29. Transfer the collected ISF into new 1.5 mL tubes, place the tubes on dry ice, and place it immediately at −80°C for long-term storage (Figure 3H).

***Note:*** Typical ISF recovery ranges from 2–4 μL for pooled naïve axillary LNs, 8–10 μL for pooled TDLN, and approximately 50 μL for around 500 mg tumor sample.

30. Optional: If desired, tissues after centrifugation can either be further processed (flow cytometry or OCT embedding) or disposed of.

***Note:*** Aliquot ISF based on downstream experiments to avoid repeated freeze-thaw cycles, as they may affect protein stability and bias detection of low-abundance proteins. Store continuously at −80°C. We recommend processing the samples within 2 years of collection.

### ISF protein digestion and analysis by mass spectrometry

**Timing: 2 days**

This section describes sample preparation, digestion, clean-up, and mass spectrometry-based analysis of ISF-derived peptides. All reagents used should be MS-grade, where available.

**CRITICAL:** Handle hazardous reagents and organic solvents, including formic acid, acetonitrile, TCEP, and 2-chloroacetamide, according to institutional chemical safety guidelines. Wear appropriate personal protective equipment and work in a chemical hood when required.

31. Prepare the 5× reduction/alkylation master mix as described in the “materials and equipment” section.
32. Reduce and alkylate proteins in ISF samples by adding a 5× reduction/alkylation master mix to achieve final concentrations of 10 mM tris(2-carboxy(ethyl)phosphine) (TCEP), 30 mM 2-chloroacetamide (CAA), and 100 mM Tris-HCl pH 8.5.
33. Heat the samples for 10 min at 95°C with 1000 rpm on a thermoshaker.
34. Digest proteins using 1.5 μg trypsin/LysC per sample for 16 h at 37°C, 1000 rpm on a thermoshaker.
35. Desalt samples by solid phase extraction using in-house-made styrenedivinylbenzene reversed-phase sulfonate (SDB-RPS) StageTips.^7^
36. Dry samples in a vacuum concentrator and resolubilize in 0.1% formic acid.
37. Measure peptide concentrations using a NanoDrop spectrophotometer and normalize across samples to ensure equal peptide injection.
38. Separate peptide samples using nanoflow reversed-phase liquid chromatography and analyze them by electrospray ionization tandem mass spectrometry on a high-resolution, accurate-mass instrument.

**CRITICAL:** We recommend operating the instrument in data-independent acquisition mode.

***Note:*** The following steps are based on using an EASYnLC 1200 UHPLC system with an in-house-made 50 cm long, 75 μm inner diameter column, packed with 1.9 μm ReproSil C18 beads, and an Orbitrap Exploris 480 mass spectrometer.

39. Per sample, inject 300 ng of peptide and separate at a flow rate of 300 nL/min at 55°C.
40. Elute peptides with a binary buffer system (buffer A and buffer B) and a nonlinear gradient starting at 3% buffer B and increasing to 98% buffer B over two hours.
41. Perform mass spectrometric analyses using instrument-specific software and data-independent acquisition:
  a. Acquire full MS scans over an m/z range of 300–1650 (AGC target 3 × 10^6^, maximum injection time 60 ms, resolution 120,000 at m/z 200),
  b. Acquire 32 subsequent MS/MS scans (normalized collision energy 27%, AGC target 1 × 10^6^, maximum injection time 54 ms, resolution 30,000 at m/z 200).

### Proteomics raw data preprocessing

**Timing: 1 day**

This section describes processing of data-independent acquisition mass spectrometry (DIA-MS) raw files, and suggestions for downstream filtering and imputation of missing values for proteomic analysis.

42. Process raw MS data with DIA-NN (version 1.8, https://github.com/vdemichev/diann):
  a. Enable FASTA digest for library-free search.
  b. Enable deep learning-based prediction of spectra, retention times (RTs), and ion mobilities (IMs).
43. Set the search parameters as follows, keeping all others at their default values:
  a. Set the precursor so that FDR = 1%.
  b. Set the precursor ion range to 300–1650 m/z.
  c. Set the fragment ion range to 200–1650 m/z.
  d. Enable “--relaxed-prot-inf” option through the command line.
44. Enable MBR and set the quantification strategy to “robust LC (high accuracy)”.
45. Match the spectra against the current mouse UniProt FASTA database.

***Note:*** We recommend using RT-dependent cross-run normalization implemented in DIA-NN and enabled by default. Downstream analyses should be performed using the normalized protein-group quantities reported in the protein group–centric output file, “report.pg_matrix.tsv”, or equivalently in the “PG.MaxLFQ” column of the main report, filtered using a 1% global protein-group FDR and 5% run-specific protein-group FDR (Lib.PG.Q.Value <= 0.01 and PG.Q.Value <= 0.05).

46. Filter quantified protein groups to retain those with at least 70% valid values within at least one experimental group.

***Note:*** Extracellular proteins may be further selected based on extracellular Gene Ontology cellular component annotations, including ‘extracellular region’ and ‘extracellular matrix,’ and/or the UniProt ‘Secreted’ keyword.

47. Impute the remaining missing values using a left-censored approach:
  a. Draw random values from a normal distribution with a width of 0.3 and a downshift of 1.8 standard deviations relative to the distribution of measured values.
  b. Perform the imputation in the Perseus software platform.^8^

***Note:*** Alternative imputation methods may be used depending on the data structure and analysis objectives.

### Downstream bioinformatic analysis and visualization

**Timing: 1 day**

This section describes statistical analysis and visualization of proteomics data, including differential testing, volcano plots, UMAP, and heatmap generation. A reproducible R script template for this downstream analysis is publicly available via Zenodo (https://doi.org/10.5281/zenodo.21293916).

**Figure 4:**
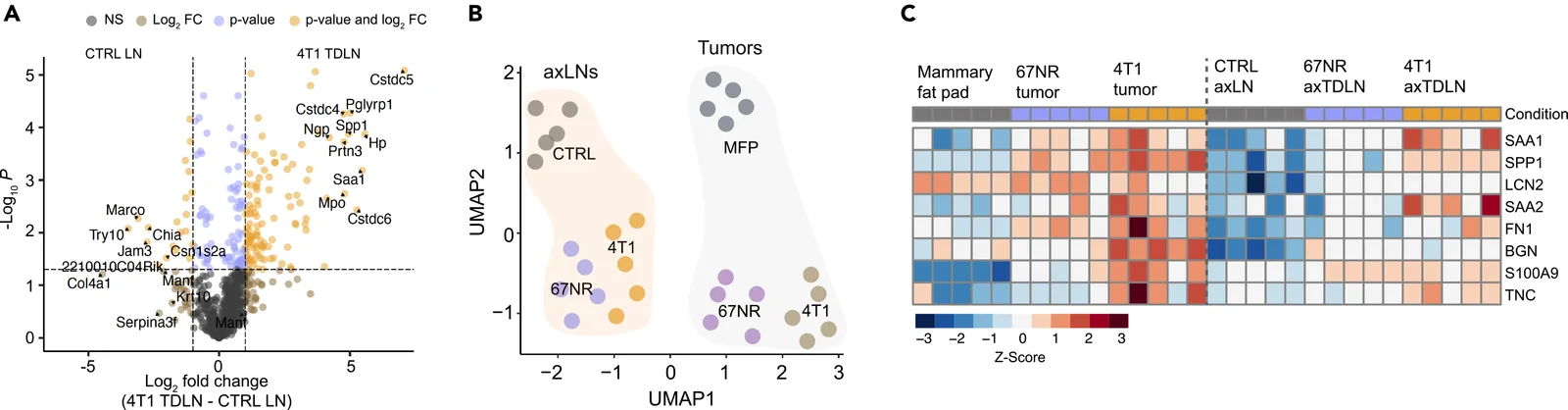
Visualization of processed proteomics data (A) Volcano plot showing the differential abundance analysis between 4T1 TDLNs and control LNs ISF samples. The dashed vertical lines indicate the log_2_ fold-change thresholds, and the dashed horizontal line indicates the adjusted p-value threshold used to define significantly altered proteins: |log_2_ fold change| ≥ 1 and adjusted p-value ≤ 0.05. (B) UMAP visualization of ISF proteomic profiles from axLNs and tumors, colored by experimental condition and tissue type. (C) Heatmap showing z-scores of selected protein abundances across mammary fat pad, tumor, CTRL axLN, and TDLN ISF samples. CTRL LN, control lymph node; TDLN, tumor-draining lymph node; MFP, mammary fat pad; 4T1 and 67NR, murine breast cancer cell lines. All example analyses presented here are adapted and the figure reprinted with permission from Mattavelli et al.^1^
  b. Define significantly altered proteins as |log_2_ fold change| ≥ 1 and adjusted p-value ≤ 0.05.
50. Perform Uniform Manifold Approximation and Projection (UMAP) on scaled protein abundance values to explore global sample relationships in a non-linear embedding.
51. Compute UMAP using the UMAP R package (v0.2.10, https://github.com/tkonopka/umap) and generate visualizations using the ggplot2 R package (v3.3.6, https://ggplot2.tidyverse.org) (Figure 4B).

***Note:*** The UMAP here is used to assess global sample variance and grouping.

48. Identify the groups to compare and perform statistical analysis using two-tailed t-tests and adjust p-values using the Benjamini–Hochberg (BH) method.
49. Generate volcano plots using the EnhancedVolcano R package (v1.22.0, https://bioconductor.org/packages/release/bioc/html/EnhancedVolcano.html):
  a. Plot log_2_ fold-change values against -log_10_ adjusted p-values (Figure 4A).

52. Generate heatmaps using scaled protein abundance values to visualize expression patterns across samples and highlight similarities and differences between groups (Figure 4C).

## Expected outcomes

This centrifugation-based method enables robust isolation of ISF from solid tissues for downstream proteomic analysis. The approach yields sufficient material for mass spectrometry while minimizing contamination from intracellular compartments. This enables reliable profiling of the extracellular proteome.

**Figure 5:**
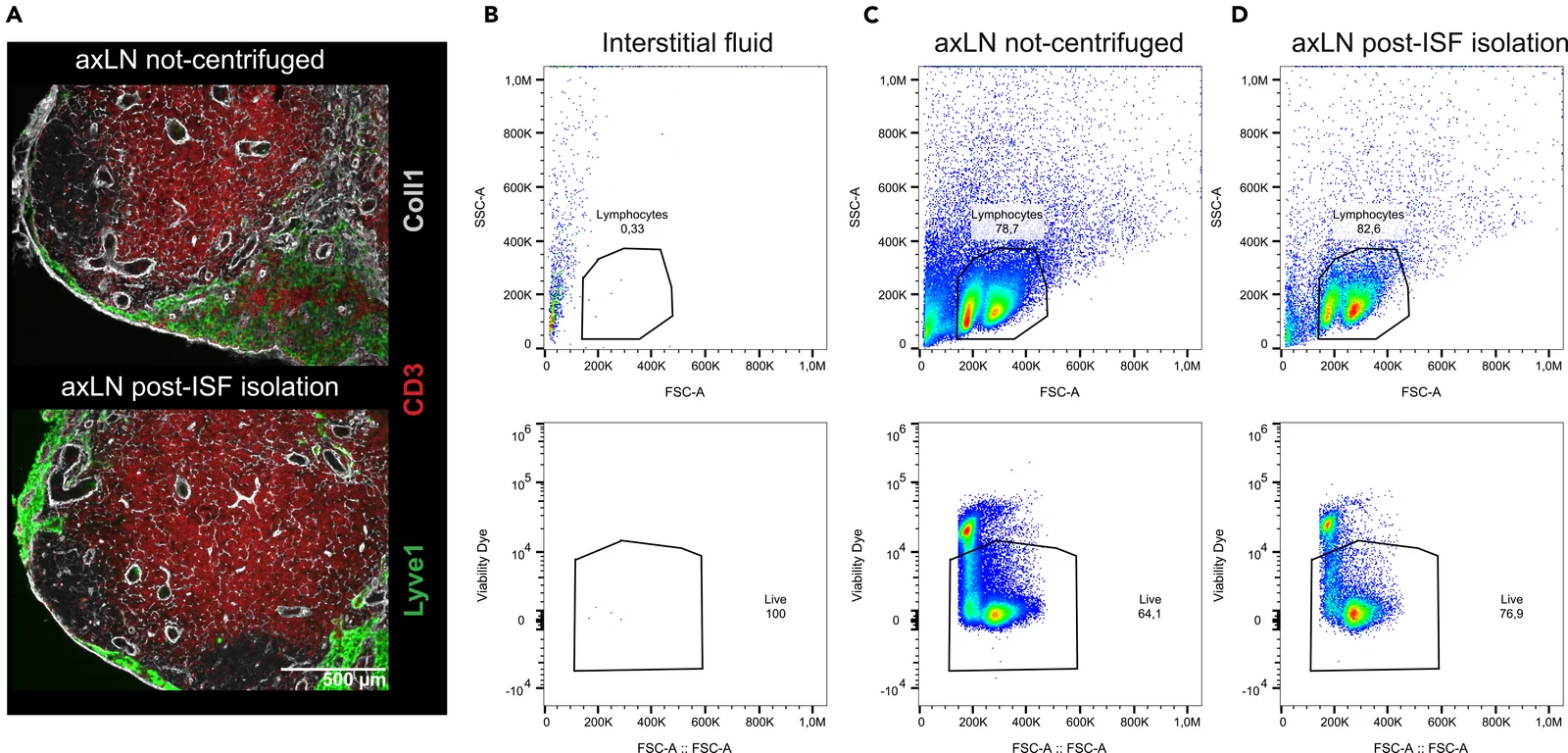
Quality-control assessment of ISF purity and lymph node integrity after ISF isolation (A) Representative immunofluorescence images of axLNs not centrifuged and post-ISF isolation. Sections were stained for Collagen I (grey), CD3 (red), and LYVE1 (green). Scale bar: 500 μm. (B–D) Representative flow cytometry plots of the recovered ISF fraction (B), axLN tissue not centrifuged (C), and axLN tissue post-ISF isolation (D). Events were visualized by FSC-A/SSC-A, and viability dye staining was used to identify live cells. Numbers indicate the percentage of events within the indicated gates.

Importantly, low-speed centrifugation preserves tissue viability and maintains compatibility with subsequent tissue-based analyses. To evaluate tissue viability and preservation of tissue architecture after ISF extraction, remaining tissues were analyzed by flow cytometry and immunofluorescence staining. After centrifugation, cell viability remained unaltered in LNs, and immunofluorescence staining showed that tissue structure integrity was preserved (Figures 5A–5D). This supports the use of the recovered tissues for downstream applications.

Application of this protocol is intended to generate reproducible, tissue-specific proteomic signatures reflecting primary tumor-driven and LN-associated microenvironmental remodeling. In this context, TDLNs are predicted to have different proteomic profiles than naïve LNs as they undergo tumor-induced remodeling at the pre-metastatic stage.

Additionally, differences in proteomic remodeling are also expected between tumor models with distinct metastatic properties, such as 4T1 and 67NR. These differences reflect their distinct tumor aggressiveness and capacity to reshape both the primary tumor microenvironment and the draining LN niche.

Overall, this approach is designed to identify extracellular proteins that are either derived from or induced by tumors and shared between primary tumors and TDLNs. This enables the identification of model-specific proteins for tumor-driven remodeling and pre-metastatic niche formation.

## Quantification and statistical analysis

Statistical analysis was performed using two-tailed t-tests with p-value adjustment by the Benjamini-Hochberg method. Fold-change values and adjusted p-values were used for comparative analyses and visualization, including volcano plot generation.

## Limitations

Although the approach for isolating ISF and performing downstream proteomic profiling is robust, several limitations should be considered when interpreting the data.

The extracellular proteome is often dominated by highly abundant, plasma-derived proteins such as immunoglobulins and albumin. These proteins can mask low-abundance proteins, including cytokines and chemokines. Complementary targeted approaches, such as multiplex immunoassays or ELISA-based platforms (e.g., LegendPlex), are necessary to detect these proteins. Furthermore, proteolytic activity during tissue dissection can lead to the partial degradation of unstable extracellular proteins. While rapid processing on ice minimizes this effect, it does not fully prevent time-dependent proteolysis.

Although low-speed centrifugation minimizes mechanical tissue disruption, contamination from intracellular proteins cannot be completely prevented, especially due to the release of intracellular contents from apoptotic or fragile cells during processing. For this reason, downstream analyses should include strict filtering of extracellularly annotated proteins to minimize contamination from intracellular sources. Distinguishing processing-induced protein leakage from intrinsic baseline cell turnover in LN tissue would require a dedicated calibration study comparing high-quality ISF with deliberately mishandled samples and remains a priority for future protocol refinement.

Additionally, this method only provides a snapshot of the extracellular proteome at one point in time. Therefore, selecting the appropriate time point for the experiment is essential, whether early or late remodeling processes are to be investigated. This protocol was optimized for the analysis of pre-metastatic axillary TDLNs, metastatic LNs may display different ISF composition and should be evaluated separately. Furthermore, recovering ISF from LNs necessitates the addition of PBS. As a result, the recovered material is diluted extracellular fluid, so absolute protein concentrations may not accurately reflect native extracellular concentrations.

Also, this method does not preserve spatial information within tissues because ISF provides a general overview of the extracellular compartment. Therefore, this approach cannot identify region-specific proteomic heterogeneity in tumors or LNs.

Finally, the yield of ISF from LNs is typically low. This can limit the number of possible downstream applications and can require larger sample sizes or pooling strategies to obtain sufficient material for proteomic analysis.

## Troubleshooting

### Problem 1

Technical variability in orthotopic injection (related to Step 12).

### Potential solution

To ensure the accuracy of orthotopic injections, it is essential to have a precise understanding of the technique, which requires dedicated training and practice to achieve correctness and consistency. In the case of the 4^th^ mammary fat pad, cells can begin to grow in the peritoneum of the mouse. This indicates either an inappropriate injection into the fat pad or leakage of the cells. To avoid this, ensure that a bubble of cell suspension is formed during injection, and quickly dry any leakage to prevent tumor cells from spreading to the peritoneum.

### Problem 2

Heterogeneous tumor growth (related to Step 13).

### Potential solution

Always verify cell viability and concentration using an automated cell counter prior to injection. Also, ensure that the number of tumor cells being injected is optimized. Low cell numbers may result in tumor rejection, while high cell numbers may lead to excessively rapid tumor growth.

### Problem 3

Insufficient ISF volume (related to Step 28).

### Potential solution

Typical ISF recovery from naïve axillary LNs ranged from 2–4 μL per pooled sample, whereas TDLNs yielded 8–10 μL, depending on the size. Tumor samples of approximately 500 mg typically yielded 50 μL ISF. If the volume recovered after centrifugation is too low, make sure the centrifuge is set to 4°C and the room temperature is 21°C. When working in rooms with higher temperatures, cover the filter and tissue with parafilm to reduce evaporation. If the ISF yield remains insufficient, increasing the number of pooled LNs or biological replicates is recommended. Increasing the PBS volume is not recommended, as this may further dilute extracellular proteins and affect quantitative comparisons.

### Problem 4

High variability between replicates (related to Step 48).

### Potential solution

Variability may arise from inconsistent ISF yield or timing of dissection. Therefore, it is essential to standardize dissection timing and strictly adhere to centrifugation parameters and storage procedures.

## Resource availability

### Lead contact

Further information and requests for resources and reagents should be directed to and will be fulfilled by the lead contact, Angela Riedel (angela.riedel@uni-wuerzburg.de).

### Technical contact

Technical questions on executing this protocol should be directed to and will be answered by the technical contact, Greta Mattavelli (greta.mattavelli@uni-wuerzburg.de).

### Materials availability

This study did not generate new, unique reagents.

### Data and code availability

The mass-spectrometry proteomics data have been deposited to the ProteomeXchange Consortium (http://proteomecentral.proteomexchange.org) via the PRIDE partner repository with the dataset identifier PRIDE: PXD066773. The reproducible R-script template for the bioinformatic downstream analysis is available via Zenodo (https://doi.org/10.5281/zenodo.21293916).

## Acknowledgments

This work was supported by funding from the 10.13039/501100005972German Cancer Aid (MSNZ Würzburg/NG2 to A.R.), from the IZKF Würzburg (project C-524 to A.R.), and from the 10.13039/501100003042Else Kröner-Fresenius-Stiftung (2022_EKEA.76 to A.R.). G.M. and M.H. were supported by funds from the Bavarian State Ministry of Science and the Arts and the 10.13039/501100020613Graduate School of Life Sciences (GSLS) of the University of Würzburg. The graphical abstract was created using images generated by Marion Krafft.

## Author contributions

A.R. conceived the study, designed experiments, and interpreted data. G.M. performed the majority of experiments and analyses. M.H. performed all bioinformatics analysis. J.J.S. and F.M. performed proteomics analysis. G.M., M.H., J.J.S., A.A. and A.R. prepared the figures and wrote the manuscript. A.R. supervised the study and acquired funding. All authors contributed to editing of the manuscript and critical review.

## Declaration of interests

The authors declare no competing interests.

## Declaration of generative AI and AI-assisted technologies in the writing process

During the preparation of this work, the authors used DeepL and ChatGPT for language and grammar editing. After using these tools, the authors reviewed and edited the content as needed and take full responsibility for the content of the publication.
