# Protocol for isolation of interstitial fluid from tumor-draining lymph nodes for proteomic profiling in murine breast cancer models

**DOI:** 10.1016/j.xpro.2026.104852

**Published:** 2026-09-08

**Authors:** Greta Mattavelli, Moutaz Helal, Jonathan J. Swietlik, Arpa Aintablian, Felix Meissner, Angela Riedel

## Main text

(STAR Protocols *7*, 104816; September 18, 2026)

In the originally published version of this article, the author list mistakenly appeared out of order; specifically, Moutaz Helal appeared later in the author list than intended. The author list has now been corrected online. This error occurred during production due to an oversight by production staff and the authors. Cell Press and the authors apologize for the mistake.

